# Elevational patterns of *Polylepis* tree height (Rosaceae) in the high Andes of Peru: role of human impact and climatic conditions

**DOI:** 10.3389/fpls.2014.00194

**Published:** 2014-05-09

**Authors:** Michael Kessler, Johanna M. Toivonen, Steven P. Sylvester, Jürgen Kluge, Dietrich Hertel

**Affiliations:** ^1^Institute of Systematic Botany, University of ZurichZurich, Switzerland; ^2^Department of Biology, University of TurkuTurku, Finland; ^3^Faculty of Geography, University of MarburgMarburg, Germany; ^4^Plant Ecology and Ecosystems Research, Albrecht von Haller Institute for Plant Sciences, University of GöttingenGöttingen, Germany

**Keywords:** air temperatures, forest structure, soil temperatures, solar radiation, alpine treeline, tropical forest

## Abstract

We studied tree height in stands of high-Andean *Polylepis* forests in two cordilleras near Cuzco (Peru) with respect to variations in human impact and climatic conditions, and compared air and soil temperatures between qualitatively defined dry and humid slopes. We studied 46 forest plots of 100 m^2^ of five *Polylepis* species at 3560–4680 m. We measured diameter at breast height (dbh) and tree height in the stands (1229 trees in total), as well as air and soil temperatures in a subset of plots. The data was analyzed combining plots of given species from different sites at the same elevation (±100 m). There was no elevational decrease of mean maximum tree height across the entire data set. On humid slopes, tree height decreased continuously with elevation, whereas on dry slopes it peaked at middle elevations. With mean maximum tree heights of 9 m at 4530 m on the humid slopes and of 13 m at 4650 m on the dry slopes, we here document the tallest high-elevation forests found so far worldwide. These highest stands grow under cold mean growing season air temperatures (3.6 and 3.8°C on humid vs. dry slopes) and mean growing season soil temperatures (5.1 vs. 4.6°C). Mean annual air and soil temperature both decreased with elevation. Dry slopes had higher mean and maximum growing season air temperatures than humid slopes. Mean annual soil temperatures did not significantly differ and mean annual air temperatures only slightly differed between slopes. However, maximum air temperatures differed on average by 6.6 K between dry and humid slopes. This suggests that the differences in tree height between the two slopes are most likely due to differences in solar radiation as reflected by maximum air temperatures. Our study furthermore provides evidence that alpine *Polylepis* treelines grow under lower temperature conditions than global high-elevation treelines on average, suggesting that *Polylepis* species may have evolved special physiological adaptations to low temperatures.

## INTRODUCTION

The tropical Andes support some of the World’s highest forests, mainly formed by species of the genus *Polylepis* (Rosaceae). With about 30 species (Kessler and Schmidt-Lebuhn, 2006), this tree genus has radiated into a wide range of ecological niches, ranging from very wet cloud forests to high-elevation semideserts (Simpson, 1986; Schmidt-Lebuhn et al., 2010). Two features have rendered *Polylepis* prominent in the literature of tropical high mountain ecosystems. First, *Polylepis* forms one of the highest alpine treelines worldwide, with 3 m tall trees recorded at 4810 m on Volcán Sajama in Bolivia (Hoch and Körner, 2005), only surpassed by similarly tall *Juniperus tibetica* at 4900 m in Tibet (Miehe et al., 2007). Second, in much of the tropical Andes, the distribution of *Polylepis* forests is disconnected from that of other montane forests at lower elevations. Accordingly, the upper limit of the closed montane forest belt found at elevations between 3200 and 3800 m is often considered to represent the natural alpine treeline (Gosling et al., 2009; Urrego et al., 2011), while it remains debated to what degree the disjunct and patchy distribution of *Polylepis* forests, with stands often being restricted to special microsites, is natural or man-made (e.g., Kessler, 2002; Gosling et al., 2009; Urrego et al., 2011; Gareca et al., 2013). Nevertheless, there is no doubt that many *Polylepis* forest stands are currently strongly affected by human activities, either directly by timber extraction, or indirectly by cattle grazing and associated grassland burning (Kessler, 2000, 2002; Renison et al., 2006, 2010). It has been shown that these activities influence forest density and height (Toivonen et al., 2011), tree regeneration (Cierjacks et al., 2007; Bader and Ruijten, 2008), and the genetic constitution of *Polylepis* populations (Hensen et al., 2012; Gareca et al., 2013). In this context, understanding how climatic conditions, as well as human activities, affect the growth of *Polylepis* trees is of considerable interest, both with regard to the ecology of high-elevation forest ecosystems, and as a baseline for the management of natural tree resources.

Tree height is one of the most meaningful ecological variables regarding the growth performance of trees, yet its spatial variability remains incompletely documented and understood, both for the tree growth form in general (Cramer, 2012) and for *Polylepis* trees in particular (Kessler et al., 2007). While it is well known that, in general terms, tree height decreases with decreasing temperatures, water availability, and nutrient availability, these relationships are not necessarily linear and may also differ between taxa and geographical regions. In mountains, tree height generally decreases with increasing elevation (e.g., Wilson et al., 1987; Young, 1993; Kessler et al., 2007), but, especially near the upper (alpine) treeline, a wide range of tree height-elevation relationships can be found (Holtmeier, 2009; Körner, 2012). Some studies have documented linear decreases with elevation (e.g., Paulsen et al., 2000; Kessler et al., 2007), while others have found increasingly steep declines in tree height close to treeline elevation (e.g., Barrera et al., 2000; Hertel and Wesche, 2008; Hertel and Schöling, 2011b) or even abrupt treelines (e.g., Miehe et al., 2007). Furthermore, treelines may be formed by closed stands of trees or by increasingly scattered tree individuals (Miehe et al., 2007; for a review of treeline physiognomy see Holtmeier, 2009). These different treeline forms may, among other factors and depending on spatial scale, be caused by natural factors such as topography, water availability, and disturbance history as well as by human impact (Holtmeier, 2009; Quesada et al., 2009; Harsch and Bader, 2011; Cramer, 2012).

The relationship of humidity and elevation in influencing tree growth is also complex, since the precipitation regime does not necessarily show a linear elevational pattern, but is intimately dependent on the elevational position of the cloud base and the available source of water (Barry, 1992). While, generally speaking, tree height increases with increasing water availability (except in water-saturated soils; Koch et al., 2004; Oldham et al., 2010), in high mountains this effect may be overridden by low temperatures. Thus, trees may grow taller on warm, sunny slopes than on cold, shady ones even if the latter are more humid (Kessler et al., 2007; Holtmeier, 2009; Paulsen and Körner, 2014). For example, *Polylepis tarapacana* forests, at the aridity limit of genus in the high Andes of southwestern Bolivia, do not grow on the more humid but less sunny southern slopes, and instead are restricted to the dryer and more sunny northern ones (Kessler, 1995b; Kessler et al., 2007).

A further challenge in exploring structural forest parameters at treeline elevations is that human impact has affected treelines worldwide via timber extraction, burning and use as grazing lands for domestic animals (Laegaard, 1992; Kessler, 1995b, 2000; Hemp, 2005; Toivonen et al., 2011; Schüler et al., 2012). Human impact can often be detected in structural parameters of forest stands, such as by a lack of saturation of tree height with increasing stem diameter, i.e., a lack of decreasing tree slenderness. In mature, natural forests, the tree height-dbh (diameter at breast height) relationship usually shows a saturation effect because tree height is limited by ecological factors such as water and nutrient availability, or the lack of adequate thermal conditions (Anfodillo et al., 2012; Lines et al., 2012; Marshall et al., 2012). Trees that have reached this potential maximum height cannot grow any taller but, as they increase in age, they continue to form new annual growth rings, thus increasing in diameter (Domic and Capriles, 2009). By contrast, in disturbed forests where especially large trees are extracted, no such saturation is visible in the remaining tree cover.

The functional causes determining the decrease of tree height with elevation, as well as the upper limit of tree growth, are still debated (see, e.g., contradictory opinions stated by Miehe et al., 2007; Holtmeier, 2009; Körner, 2012). There is no question that, leaving aside specific local conditions, the location of the upper treeline is closely linked to low temperatures. In their global reviews of treeline positions, Körner and Paulsen (2004) and Paulsen and Körner (2014) found that the mean soil temperature of the growing season, defined by them as the period with soil temperatures at 10 cm depth consistently above 3.2°C, averages 6.4–6.7°C at alpine treeline positions worldwide, and can be used to infer mean air temperatures. Although some of the tropical sites included in their studies had somewhat lower values (4.5–5.6°C), on-site measurements at Andean sites suggest that values could even be in the range of 3–4°C (Kessler and Hohnwald, 1998; Bendix and Rafiqpoor, 2001; Hoch and Körner, 2005; Hertel and Wesche, 2008). However, since these were mostly short-term measurements, their validity remains to be confirmed. It also remains unclear how these low temperatures physiologically limit tree growth, and whether low soil or air temperatures are decisive for treeline formation. Hampered tree growth at high elevations may be related to both temperature minima (e.g., lethal frost) or maxima (via annual thermal sum). Physiologically, tree growth may be limited, e.g., by thermal constraints in tissue formation due to the strong aerodynamic coupling of trees to the atmosphere (e.g., Wilson et al., 1987; Körner, 2012) or limited water and nutrient uptake by the fine root system (Leuschner et al., 2007; Hertel et al., 2008; Hertel and Schöling, 2011a).

In the present study, our aim was to study elevational patterns of tree height among five species of *Polylepis* in relation to human impact and climatic conditions in the Cordilleras Vilcanota and Vilcabamba, Cuzco, Peru. We tested the following specific hypotheses:

(1) Tree height is lower in elevation belts where forest stands are affected by humans, compared to elevation belts where the stands are in natural conditions.

(2) Tree height is lower on humid slopes than on drier slopes because of cloudiness and decreased solar radiation and thus lower temperatures.

## MATERIALS AND METHODS

### STUDY AREA

**FIGURE 1:**
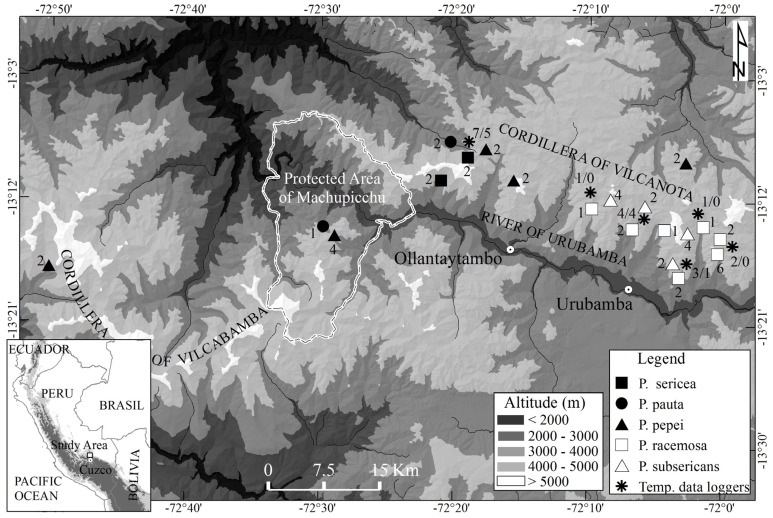
**Map of the study region, showing the location of the study sites and the temperature data loggers.** The number of plots and temperature data loggers (air/soil) in each site is indicated next to the symbols.

We carried out the field work in the Cordilleras Vilcanota and Vilcabamba, Cuzco, Peru (13°07’–13°17’ S and 72°02’–72°29’ W) for seven years between 2006 and 2012 (**Figure 1**). The Cordilleras Vilcanota and Vilcabamba are known to support some fairly extensive, and partly well preserved *Polylepis* forests at elevations between 3300 and 4950 m (Lloyd and Marsden, 2011; Toivonen et al., 2011). The climate of the region varies from humid to semi-arid with a clear wet season from November to April. Diurnal temperature fluctuations are pronounced, especially in the dry season. There is a strong geographical gradient in precipitation across the study area, caused by specific orography directing winds and cloud movements. This is shown by the contrasting precipitation records at the climate stations of Urubamba (2863 m), with 454 mm mean annual precipitation, and Wiñaywayna, protected area of Machupicchu (2800 m), with 1606 mm (records of former INRENA, National Institute of Natural Resources of Peru and SENAMHI, National Service of Meteorology and Hydrology of Peru). Based on this strong precipitation gradient and, consequently, strikingly different vegetation composition, we separated the studied sites as either dry or humid ones. The separation was mostly qualitative and the *Polylepis* species found at the different sites were used as a first indicator of the prevalent humidity conditions based on the known ecological distribution of the species (according to e.g., Kessler, 1995a,b, Fjeldså and Kessler, 1996). On the humid sites, *Polylepis* trees were covered in mats of epiphytic bryophytes, whereas on the dry sites there were noticeably less bryophytes. Moreover, as an indication of drought, the low elevation stands on dry sites are intermixed with drought-deciduous trees. The climate data from the lower elevation climate stations supports our qualitative classification.

The study sites of humid and dry areas were located on the same soil type, Regosol, according to classification of Food and Agriculture Organization of the United Nations (IMA, Instituto de Manejo de Agua y Medio Ambiente, Gobierno Regional de Cuzco, Peru, 2014). Regosols are very weakly developed mineral soils found in eroding lands, in particular in arid and semi-arid areas and in mountain regions.

In total, five *Polylepis* species are found in our study area, being segregated by elevation and humidity (Toivonen et al., 2011). Forest stands are mostly monospecific with *P. pauta* Hieron., *P. sericea* Wedd. and *P. pepei* Simpson being found in humid areas while *P. racemosa* Ruiz & Pav. and *P. subsericans* Macbr. are found in drier areas. Species have some specific climatic adaptations in their functional traits, with high-elevation species, for example, having smaller leaves and higher root tip abundance compared with lower elevation species (Toivonen et al., 2013).

The study region has been used by humans for millenia (Mosblech et al., 2012) and *Polylepis* forests, at present, are subject to different degrees of human impact (Toivonen et al., 2011). This region is, thus, ideal to study elevational trends of tree height under differing patterns of humidity and human impact.

### FIELD SAMPLING

Sampling was aimed at covering the widest possible elevational range of *Polylepis* stands while being largely determined by the availability of forest patches. In total, we studied 46 plots in *Polylepis* stands, ranging from 3560 to 4680 m, located in varying expositions. Because of the scattered distribution of forest stands, it was often impossible to find replicated, suitable forest sites at specific elevations. We, therefore, combined plots of a given species from different sites at the same elevation within elevational belts of 200 m (i.e., a deviation in elevation of ±100 m), reasoning that within-belt elevational differences are minor compared to the overall elevational gradient that we covered (3560–4680 m; **Table 1**). In total, we studied six elevational belts in humid and six in dry areas. Within each elevational belt, we established 3–6 plots of 10 m × 10 m each, with the exception of one belt where only a single suitable plot site could be located. Plots were located as far as possible in the center of the forest stands, at least 25 m from the forest edge to give a representative sample of the structure of the forest stand.

**Table 1:** Characteristics of the elevation belts and number of temperature data loggers in each belt.

Species	Mean elevation (m)	Elevation range (m)	No. of plots	Human impact	No. of individuals	Mean maximum tree height (m)^1^	No. of temperature data loggers (air/soil)
**Humid slope**
*P. pauta*	3730	3560–3760	3	No	52	14.3	2/1
* P. sericea*	3790	3725–3910	3	Yes	84	8.4	1/1
*P. pepei*	4140	4120–4195	3	Yes	108	5.8	1/1
*P. sericea*	4230	4230–4230	1	Yes	94	4.2	1/1
*P. pepei*	4370	4235–4415	6	No	164	9.2	2/1
*P. pepei*	4530	4440–4565	3	No	107	8.7	0/0
**Dry slope**
*P. racemosa*	3980	3865–4070	5	No	96	15.9	2/2
*P. racemosa*	4160	4140–4230	4	Yes	103	9.6	3/1
*P. racemosa*	4300	4280–4355	4	No	76	18.5	1/0
*P. subsericans*	4340	4270–4390	5	Yes	105	11	1/1
*P. subsericans*	4430	4410–4460	4	Yes	90	11.3	3/1
*P. subsericans*	4650	4635–4680	5	No	150	12.8	1/0

*Human impact was determined based on signs of humans (paths, cut trees, and branches) or their animals (hoofprints, feces).
*

1 Mean of 10% tallest trees.

In each plot we measured dbh and visually estimated tree height of all trees ≥10 cm of circumference at breast height. For tree height estimation, we used a 1.5 m long measuring stick placed next to each tree. The visual tree height estimation was tested for its accuracy in particular in case of taller trees by comparing the results with a standard stick method (i.e., by calculating tree height from the distance of the researcher to a stick of known height and the distance to the tree stem base according to the mathematical second intercept theorems). The results of the two methods were reasonably similar (with a maximum divergence of the results of both methods ±50 cm). We ended up by using the estimation method, because it was much more feasible to use in topographically demanding terrain. Furthermore, since the height of the trees was relatively low overall (in comparison, e.g., to mature lowland forest trees), we are rather confident that the estimates are sufficiently accurate. Because human impact is known to significantly affect *Polylepis* forest structure both in the study region (Toivonen et al., 2011) and elsewhere (Renison et al., 2006), we recorded signs of impact of human land use (cut trees, trails, fire scars) and of the domestic animals (tracks, feces, signs of grazing).

In total, we measured 1229 tree individuals of the five species of *Polylepis* (**Table 1**). The number of tree individuals measured per elevational belt varied from 52 to 164 (mean 102.4 ± 30.1 SD). Forest stands with signs of human impact (e.g., footpaths, cut trees, charcoal, feces of domestic animals, etc.) were regarded as potentially anthropogenically influenced whereas stands without such signs were regarded as not significantly affected by humans (thus representing the potential performance of the trees under natural growth conditions). In six of the elevational belts, we recorded signs of human influence whereas the other six belts were considered to be unaffected by humans (**Table 1**).

### MICROCLIMATIC MEASUREMENTS

We measured air temperature in the shaded canopy with dataloggers (DS1922 Thermochon iButtons, Hubbart et al., 2005) in 18 of our study plots and within the soil in the shaded root zone (10 cm depth) in 10 plots (**Figure 1**). Measurements were run for one, two or three years (between June 2006 and April 2012) in each plot depending on the accessibility of the stand. However, due to the problematic access, the measurements from the uppermost stands covered only the period between begin of July to end of May. However, during the whole measurement period (2006–2009) inter-annual variations in temperature were small. So in cases, where we had data from 2 to 3 years per site (most plots), we used the mean values. Measurement readings were mostly taken at 4 h intervals (starting at 00:00). In three sites, air temperature was recorded in 2 h intervals to assess whether we recorded daily minima and maxima with 4 h measuring interval at the other sites (**Table 1**). Growing season length was defined as the number of days where soil temperature at 10 cm depth is continuously above 3.2°C (Körner and Paulsen, 2004).

### DATA ANALYSES

We quantified mean maximum tree height in each elevational belt by averaging the height of 10% of the tallest trees in each belt. The relationship between mean maximum tree height and elevation was analyzed using linear regression, based on mean values per elevational belt. We tested statistically the differences in mean maximum tree height in natural stands within each slope.

In each elevational belt, we plotted tree height of each individual tree (≥10 cm of circumference at breast height) against dbh and fitted both a linear and a logarithmic function to the data points. Models were compared via their *R*^2^ and AICc-values (models with a delta AICc > 4 were claimed to be substantially better following Burnham and Anderson, 2002). As an additional potential measure of saturation of tree height, we calculated slenderness of each tree by dividing tree height by dbh (Wang et al., 1998) and compared the values between elevational belts via Analysis of Variance.

We compared temperature values between the humid and dry slopes with a *t*-test. For the temperature variables that showed a significant linear relationship with elevation, the residuals of linear regression with elevation were compared. Temperature data from both slopes were combined to extract a common trend in a decrease of temperature with elevation. The data was combined, because we did not have any *a priori* reason to expect a decrease in temperature by increasing elevation to be different between the slopes. If there was a significant trend in temperature decrease with elevation, a residual variation of temperature was compared between the slopes. If there was not a significant elevational trend in temperature, raw values were compared. In this way, the effect of elevation was taken into account before comparing the temperature conditions between the slopes, because the measurements came from different elevations. All analyses were performed with (R Core Team, 2012) and package qpcR (Spiess, 2012).

## RESULTS

### TREE HEIGHT VERSUS HUMAN IMPACT AND HUMIDITY

**FIGURE 2:**
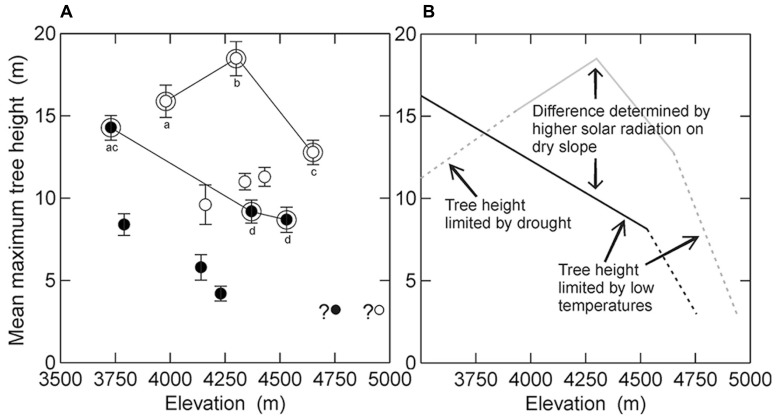
**Relationship of mean maximum tree height to elevation in the study region. (A)** Visualization of the data from this study, distinguishing between the dry (white symbols) and humid (black symbols) slopes as well as sites without evidence of human impact (double symbols linked by lines) and those with human impact (single symbols). Whiskers indicate standard deviations of the means. The two small symbols with question marks in the lower right corner correspond to the putative highest forest stands identified from aerial photographs of the study region (Toivonen et al., submitted manuscript). Different letter indicate statistically significant differences in mean maximum tree height between sites without human impact (*U* test after Mann & Whitney, *p* < 0.05). **(B)** Schematic representation of the natural pattern of tree height in the study region and the environmental factors associated with it, contrasting the dry (gray line) and humid (black line) slopes. Dashed lines correspond to expected tree height-elevation relationships based on indirect evidence (see text for details).

Analysing all twelve elevational belts together, there was essentially no elevational trend in mean maximum tree height (*R* = -0.06, *p* = 0.85). The expected negative relationship was slightly more evident, but still not significantly, when belts affected by human activities (*R* = -0.32, *p* = 0.54) were analyzed separately from those without human impact (*R* = -0.44, *p* = 0.38). Also, there were no significant elevational declines of tree height either when slopes were analyzed separately (dry slope: *R* = -0.19, *p* = 0.51; humid slope: *R* = -0.59, *p* = 0.22). Plotting mean maximum tree height against elevation showed several distinct patterns (**Figure 2A**). First, trees on the dry slope were generally considerably taller than trees at the same elevation on the wet slope (*p* < 0.05). Second, tree height at anthropogenically disturbed elevational belts was noticeably (but only marginally significantly) lower than at undisturbed elevational belts (*p* = 0.055). Third, undisturbed belts on the humid slope showed a continuous decrease of mean maximum tree height with elevation whereas on the dry slope tree height peaked at mid-elevations. This decrease in tree height was also supported by our observation of other patches of remnant vegetation without *Polylepis* at even lower elevations in the Urubamba valley (M. Kessler et al., personal observation).

**FIGURE 3:**
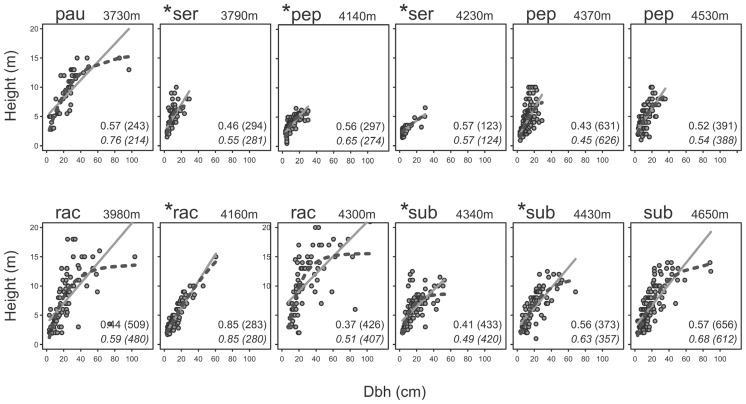
**Height-dbh relationships of *Polylepis* trees of five species at different elevational belts at the humid (upper row) and dry (lower row) sites of the Cordilleras Vilcabamba and Vilcanota.** Belts in which the studied forest stands had signs of human impact are marked by asterisks. Elevation indicates mean elevation of the plots in each elevational belt. Numbers are *R*^2^ values followed by AICc values in brackets, above for the linear models (continuous lines) and below in italics for the non-linear models (dashed lines). Species name abbreviations: pau, *pauta*; pep, *pepei*; rac, *racemosa*; ser, *sericea*; sub, *subsericans*.

Tree height and dbh were moderately to highly correlated with each other in all elevational belts, but the shapes of the relationships varied (**Figure 3**). In four belts (*pauta* 3730 m, *racemosa* 3980 and 4300 m, *subsericans* 4650 m), all of which had no signs of human impact, tree height showed a distinct saturation with increasing dbh so that the *R*^2^ values of the non-linear models were at least 0.1 higher than those of the corresponding linear models, with ΔAICc values > 19. The other belts, including two undisturbed and all six disturbed belts, showed more or less linear relationships with ΔR^2^ values between 0.00 and 0.09 and ΔAICc values between 1 and 23.

Slenderness was significantly higher at the two belts of *P. sericea* than at the other localities (one-way ANOVA, *F*_12,1404_ = 11.033, *p* < 0.001), but there were no elevational trends (linear regressions, humid slope: *R*^2^ = 0.21, *p* = 0.35; dry slope: *R*^2^ = 0.00, *p* = 0.96), nor were there visible differences between disturbed and undisturbed belts.

### TEMPERATURE CONDITIONS ON HUMID AND DRY SLOPES

**FIGURE 4:**
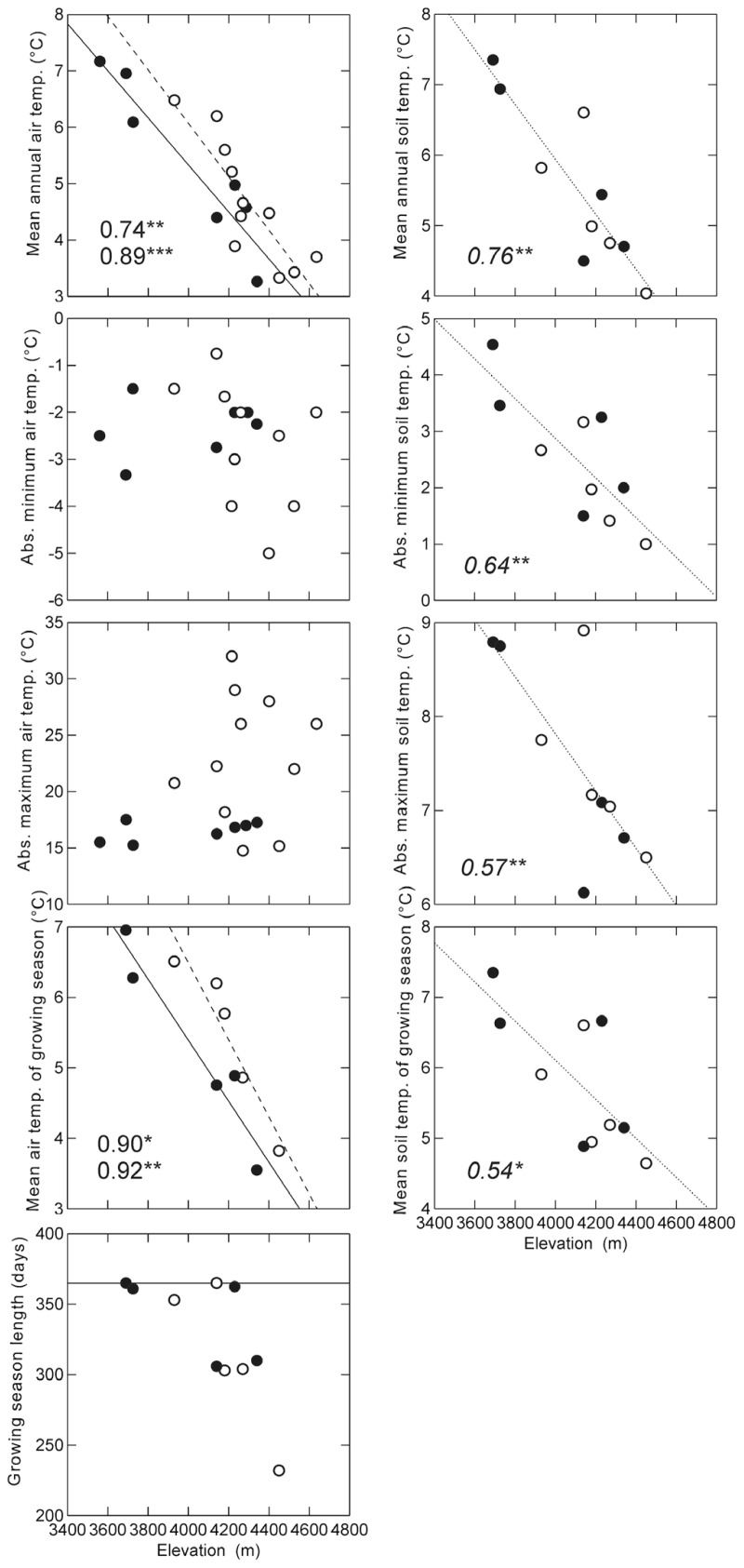
**Relationships of measured climatic variables to elevation along the dry (open symbols, dashed lines) and humid (closed symbols, continuous lines) slopes.** Growing season length was defined as the number of days with soil temperatures at 10 cm depth consistently above 3.2°C following Körner and Paulsen (2004). Lines show significant linear regression trend lines, numbers indicate the *R*^2^ values of the regression analyses (top: dry slope; bottom: humid slope). For soil temperatures, regression lines were drawn and calculated combining data from both slopes. In the graph of the growing season length, the horizontal line represents the maximum possible number of days (365) *p < 0.05; **p < 0.01; ***p < 0.001.

Overall, most temperature variables showed a decrease with elevation, with mean annual air temperature decreasing at a rate of 4.2 K per 1000 m of elevational increase on the humid slope and 4.7 K on the dry slopes (**Figure 4**). Mean air temperature of the growing season showed a steeper elevational decline both on the dry (5.5 K per 1000 m) and on the humid slope (4.3 K per 1000 m). Mean annual soil temperatures (combined for both slopes) declined at a rate of 3.9 K per 1000 m, absolute minimum temperatures at 3.5 K per 1000 m, absolute maximum temperatures at 3.0 K per 1000 m, and mean temperatures of the growing season at 2.8 K per 1000 m. Absolute maximum and minimum air temperatures showed no clear elevational patterns.

**FIGURE 5:**
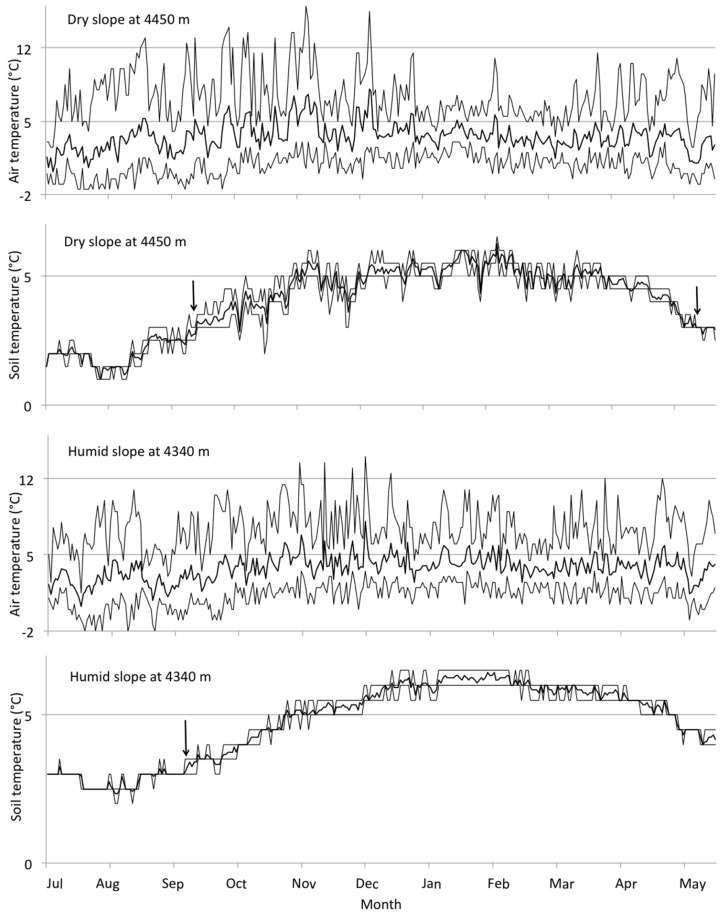
**Seasonal variation in air and soil temperatures at the highest sites in dry and humid areas in 2007–2008.** Solid lines in the middle indicate daily mean temperatures and dashed lines above and below indicate daily minimum and maximum temperatures. The beginning and the end of the growing season, based on the criteria by Körner and Paulsen (2004), is indicated with arrows. Data from June is missing, and consequently the indication of the end of the growing season at the humid site.

Considering temperature conditions at the highest sites, on the humid slope at 4330 m we measured a mean annual air temperature of 3.3°C and a mean annual soil temperature of 4.7°C, with mean air and soil temperatures for the growing season (310 days) 3.6 and 5.1°C, respectively. On the dry slope at 4450 m, mean annual air and soil temperatures were 3.3 and 4.0°C, respectively, while mean air and soil temperatures for the growing season (232 days) were 3.8 and 4.6°C, respectively (**Figure 5**). Days on which air temperature was >5°C were 34 days for dry and 36 days for humid sites. Annual thermal sums based on air temperatures were also rather similar between dry and humid sites (1124°C in dry and 1181°C in humid sites), as were annual mean air temperatures (3.3°C). Annual thermal sums based on soil temperature differed between dry and humid sites (1293°C in dry and 1502°C in humid areas, however, note that these numbers covered only a c. 11 months-long period of measurements). The definition of growing season as the number of days where soil temperature at 10 cm depth is continuously above 3.2°C (Körner and Paulsen, 2004) appeared to reflect a threshold of 0°C in daily minimum, but not daily mean air temperature (**Figure 5**).

Contrasting the temperature values of the humid and dry slopes, we found significant differences for absolute maximum air temperature and for mean air temperature of the growing season. There was also a tendency toward higher mean annual air temperatures on the dry slope, but this was only marginally significant. Mean annual soil temperatures did not differ between dry and humid slopes (**Table 2**).

**Table 2:** Comparison of temperature measurements between the dry and humid slopes in the study region.

Parameter	Dry slope	Humid slope	*t*	*p*
Residuals (mean annual air temperature)	0.13 ± 0.58	-0.33 ± 0.49	1.79	0.09
Residuals (mean annual soil temperature)	0.09 ± 0.65	-0.03 ± 0.50	0.31	0.77
Absolute minimum air temperature	-2.58 ± 1.28	-2.33 ± 0.60	-0.56	0.58
Residuals (absolute minimum soil temperature)	-0.22 ± 0.53	0.09 ± 0.81	-0.73	0.49
Absolute maximum air temperature	23.10 ± 5.65	16.52 ± 0.50	3.87	0.01
Residuals (absolute maximum soil temperature)	0.10 ± 0.73	-0.39 ± 0.58	1.18	0.27
Residuals (mean air temp. of growing season)	0.56 ± 0.45	-0.26 ± 0.37	3.17	0.01
Residuals (mean soil temp. of growing season)	-0.02 ± 0.59	0.18 ± 0.76	-0.48	0.65

*For those factors for which significant linear relationships to elevation were recovered when combining data from both dry and humid slopes, *t*-tests were used to compare the residuals of the dry against the humid sites. For those two parameters without significant elevational trends, the raw values were compared.*

## DISCUSSION

### TREE HEIGHT AND HUMAN IMPACT

At six of the elevational belts studied, we found no evidence of human impact whilst six other belts showed signs of human influence (footpaths, cut trees, charcoal and/or feces of domestic animals). Elevational belts with prevalent human activity did not show a saturation of tree height with increasing dbh. The height-dbh relationship was closer to linear in disturbed elevational belts, whereas at four of the undisturbed elevational belts, maximum tree height leveled off at a certain dbh. While very little is known about tree height-dbh relationships at high elevations, we interpret a tree height saturation with dbh as an indication of a natural tree growth pattern, where trees have reached their ecologically determined potential tree height at a given elevation (Anfodillo et al., 2012; Lines et al., 2012; Marshall et al., 2012).

Deviations from this general pattern were found at three belts dominated by *Polylepis pepei*, a relatively small species originally described as a shrub (Simpson, 1979) or tree to 3 m tall (Kessler, 1995a). Since then, a study at 3800–4050 m in Bolivia has documented trees of *P. pepei* of about 8 m height and 23 cm dbh (Hertel and Wesche, 2008) and the mean maximum tree height of 9.2 m and dbh values of up to 35.7 cm documented here at 4370–4530 m further validate that this species can become larger than previously believed. Yet, two elevational belts did not show tree height saturation despite being stands in extremely remote locations without signs of human impact and therefore regarded by us as truly undisturbed. It thus appears that *P. pepei* does not achieve a significant increase in dbh once maximum tree height has been reached, perhaps suggesting that these trees do not reach great ages. In fact, the oldest documented *P. pepei* trees are only 137 years old (Roig et al., 2001; Jomelli et al., 2012), whereas more than 700 years old *P. tarapacana* trees have been found in Bolivia (Solíz et al., 2009).

Another interesting case is presented by the stands of *P. pepei* studied in the elevational belt at 4140 m. Here, trees showed a clear height saturation, yet, with a mean maximum height of 5.8 m, were considerably lower than the stands higher up at 4370 and 4530 m, which were around 9 m tall. We believe that this is due to the fact that, at 4140 m, where human impact was fairly pronounced, the stands were restricted to very steep, rocky slopes with shallow soils on which trees could only develop stunted growth forms. Thus, although these trees apparently reached their full potential under these site conditions, their height was presumably not representative for the elevational belt as such.

### ELEVATIONAL PATTERNS OF TREE HEIGHT IN NATURAL STANDS

The highest stands studied were located at 4570 m on the humid slopes and at 4680 m on the dry slopes, but based on aerial photographs, stands of *P. pepei* on the humid slope are found at about 4700 m and of *P. subsericans* on the dry slope at 4950 m (**Figure 2A**). Although the aerial photographs do not allow us to quantify tree height, if we consider that these are the highest stands in the study region, these trees are unlikely to be more than a few meters tall. This suggests that tree height must decrease fairly abruptly above the stands found by us. This apparent abrupt decrease contradicts the simple height-elevation models applied to Bolivian *Polylepis* forests by Kessler et al. (2007) but is in accordance with height measurements of *Polylepis pepei* stands in Bolivia (Hertel and Wesche, 2008) and of other tree species at tropical treelines elsewhere (Miehe and Miehe, 1994; Miehe et al., 2007).

On the humid slope, mean maximum tree height decreased in undisturbed stands gradually from 14 m at 3730 to 9 m at 4530 m (**Figure 2A**). This constant decrease of tree height with elevation is the most commonly observed pattern of tree height-elevation relationships (e.g., Young, 1993; Paulsen et al., 2000; Kessler et al., 2007), although we cannot extrapolate this beyond the elevations studied by us. Yet, our observations in the study region also show that tree height further increases below 3700 m in mixed species forests that lack *Polylepis* trees (**Figure 2B**, M. Kessler, unpublished data). In contrast, in undisturbed stands on the dry slopes, tree height peaked at 18.5 m at 4300 m and decreased toward both lower elevations (16 m at 3980 m) and higher elevations (13 m at 4650 m).

We are thus faced with marked differences in tree height-elevation relationships on the climatically contrasting slopes of our study region, both in terms of pattern and magnitude. The hump-shaped pattern of tree height on the dry slope is difficult to interpret, but one possible explanation can be drought stress toward the valley bottom (e.g., mean annual precipitation 454 mm in Urubamba, following the Peruvian climate service SENAMHI, registration period 1963–1998). While natural vegetation is largely absent from this valley that has been under intensive cultivation for millennia, and tree height can thus not be measured below the elevations covered by us, vegetation remnants suggest that, below the lower limit of *P. racemosa* at about 3500 m, the original vegetation may have consisted of a low stature forest of *Acacia* and *Prosopis* (W. Galiano, personal communication). This thus represents an even more pronounced reduction of tree height with decreasing elevation (**Figure 2B**). Such drought-related inverse treelines have been documented, e.g., at the forest-steppe ecotone in Patagonia (Hertel et al., 2008) and are also common in rain-shadowed mountain valleys (Holtmeier, 2009). However, it is also possible that mean maximum tree height at the lowest elevation belt on dry slopes is influenced by a long-term human impact, even though we did not find recent signs of human activities in the studied stands.

At higher elevations, the marked differences in mean maximum tree height on the humid and dry slopes also begs explanation. It is well known that trees grow taller under semi-arid to sub-humid mountain conditions than in wet habitats (Kessler et al., 2007; Miehe et al., 2007; Körner, 2012; Paulsen and Körner, 2014), so that these differences are most likely a result of climatic factors such as lower solar radiation and temperatures under cloudy conditions (Lauer, 1982). Among the temperature values measured by us, we only found significant differences between the dry and humid slopes with respect to maximum and mean air temperatures. Mean air temperatures were less than 1 K higher at a given elevation on the dry than on the humid slope, whereas maximum air temperatures differed on average by 6.6 K between dry and humid slopes. Therefore, it seems that the differences in tree height between the two slopes are most likely due to differences in solar radiation as reflected by maximum air temperatures. While our measurements of within-canopy air temperatures can only be taken as rough indications of the actual temperature of the foliage, our results nevertheless show that tree height in the study region is more closely related to air and presumably above-ground tissue temperatures than to soil temperatures, which did not differ markedly between dry and humid slopes. Aspect- and, hence, radiation-related differences in tree height have also previously been documented in *Polylepis* forests in Bolivia (Kessler et al., 2007). Further, it has been shown that maximum photosynthesis, and therefore possibly tree growth, is limited by cloud cover in Amazonian cloud forest trees, emphasizing the importance of solar radiation on tree growth (Letts and Mulligan, 2005). However, a negative effect of solar radiation on biomass production has also been documented in humid cloud forests with trees investing more in biomass production in the cooler season with lower solar radiation and more in maintenance during the warmer and high solar radiation period (Girardin et al., 2014).

Comparisons of the annual mean and maximum temperatures between the sites where temperatures were recorded in 2 h intervals (one site on the humid and one on the dry slopes) and sites where temperatures were recorded in 4 h intervals, showed that, specifically on dry slopes, logging interval may have affected daily air temperature maxima, with lower values being registered for 4h interval recordings in comparison with 2 h interval recordings. This means that real values for annual mean and maximum air temperatures on the dry slopes would be higher than we measured. On the humid slope, the difference in the air temperatures between the measurement carried out in 2 and 4 h intervals was marginal (<0.5 K). However, even with this possible measurement error, the overall differences in air temperatures between the slopes, with dry slopes being warmer than humid slopes, our results provide support for the role of air temperatures in explaining the difference in tree height between dry and humid slopes.

Although most of our interpretation of differences in tree height between the dry and humid slopes focuses on climatic differences, it should be borne in mind that other factors may also play a role. First, it is conceivable that local site factors such as microtopography and soil conditions, which are well known to influence tree height (Koch et al., 2004; McNab, 2010; Unger et al., 2012), might have influenced the patterns found by us. However, we do not consider this to be the case since the large number of study plots (46 in total) and the replicates in each elevation belt exclude the impact of single site conditions on our overall findings. Furthermore, we did not find any significant differences in the topographic positions of plots on the different slopes, e.g., with regard to inclination, distance from streams, or distance to rock faces (J. Toivonen et al., unpublished data). The vast majority of plots were located within the same soil class (IMA, Instituto de Manejo de Agua y Medio Ambiente, Gobierno Regional de Cuzco, Peru, 2014). However, it is likely that humid and dry soils have consistent differences due to the climatic influence on soil development (Curtis, 1990). Another possible reason for the differences in tree height might be related to the tree species involved, which differed between the slopes. Testing this possibility would require reciprocal transplantations between the slopes, which might be problematic because it is likely that the trees would not survive, or have an altered growth, under the “wrong” climatic conditions. While such explanations may have some affect on tree growth, we still consider climate to be the most likely driver of differences in tree height between the slopes. These climate-related differences in tree growth have also been found in many other geographical areas for a wide range of taxa (Kessler et al., 2007; Miehe et al., 2007; Körner, 2012; Paulsen and Körner, 2014).

### ARE *POLYLEPIS* FORESTS FOUND UNDER LOWER TEMPERATURE CONDITIONS THAN OTHER TREELINE FORESTS?

The highest stands where microclimatic measurement were carried out on humid slopes (*P. pepei* at 4330 m) had mean soil temperatures for the growing season of about 5.1°C, and the highest stands on dry slopes (*P. subsericans* at 4450 m) of about 4.6°C. These temperatures are close to those reported from *Polylepis* treeline positions elsewhere in the Andes, e.g., mean soil temperatures of the growing season of 4.5–6.0°C at 4000–4100 m in Ecuador (Lauer and Rafiqpoor, 2000, 2002) and of 4.7–5.4°C at 4810 m in western Bolivia (Hoch and Körner, 2005). Other, more short-term measurements show even lower soil temperatures at *Polylepis* treelines in the range of 3–4°C (Kessler and Hohnwald, 1998; Hertel and Wesche, 2008). There is, thus, increasing evidence that high-elevation forests, formed by species of the genus *Polylepis* in the Andes, are found under lower temperature conditions, especially of soil temperatures, than the global mean for high-elevation treeline forests (Körner and Paulsen, 2004; Körner, 2012). There may be various reasons for this. First, as discussed above, it is unclear whether soil temperatures are a truly physiologically limiting factor for tree growth at treeline elevations. Indeed, our study suggests that air temperatures may be physiologically more important due to direct atmospheric coupling of high tree stature. It has been shown that apical meristems of high stature vegetation face lower temperatures than those of short vegetation (e.g., Hadley and Smith, 1987; Wilson et al., 1987; Grace et al., 1989; Körner, 2003). Thus, it is conceivable that *Polylepis* treelines are determined by the same temperature threshold as other treelines, but that this is not reflected by the mean soil temperatures of the growing season. Second, temperature measurements at some other tropical treelines may have been conducted at locations below the potential upper limits of tree growth. For example, the high mean soil temperatures of the growing season of 7.4°C at 3740 m on Mt. Kinabalu, Borneo (Körner, 2012), likely reflect the fact that the rain-swept rocky dome of this mountain lacks soil deep enough for tree growth, resulting in a lowered treeline. Third, species of the genus *Polylepis* may have special functional adaptations to low temperatures that enable them to grow at higher elevations than other tropical treeline species. These adaptations could include decreased leaf size and carbon assimilation rate but higher root tip abundance as found among species of *Polylepis* growing at high elevations compared to species from lower elevations (Toivonen et al., 2013). This reflects the gradual adaptation of the genus from the ancestral habitat in humid cloud forests to increasingly dry and cold conditions (Simpson, 1986; Schmidt-Lebuhn et al., 2006, 2010). Future research directed at identifying the physiological bases of these adaptations, ideally in an evolutionary context, may not only reveal how *Polylepis* has managed to colonize extremely stressful environments, but may also provide insights into the physiological limitations of tree growth at high elevations in general.

## CONCLUSION

We studied the role of human impact and climatic conditions on tree height among five species of the high Andean *Polylepis* tree genus. With a mean maximum tree height of 9 m on the humid slopes at 4530 and of 13 m at 4650 m on the dry slopes, we document taller trees than in any previous studies carried out at similar elevations worldwide (Hoch and Körner, 2005; Kessler et al., 2007; Miehe et al., 2007; Bader and Ruijten, 2008). We confirm our hypotheses that tree height is lower in the stands affected by human impact than in the stands in natural conditions, and that tree height is lower on humid than on dry slopes. We also show that humid slopes are colder than dry slopes with respect to air temperatures. Taking into account tree height differences between humid and dry slopes, this suggests that air temperatures are a decisive factor in limiting tree growth at high elevations. Additionally, we show that *Polylepis* treeline forests grow under lower temperature conditions than the global mean for high-elevation treeline forests (Körner and Paulsen, 2004).

## Conflict of Interest Statement

The authors declare that the research was conducted in the absence of any commercial or financial relationships that could be construed as a potential conflict of interest.
